# Predictors of dronedarone plasma drug concentrations and effect on atrial fibrillation/atrial flutter recurrence: Analyses from the EURIDIS and ADONIS studies

**DOI:** 10.1002/clc.23768

**Published:** 2022-01-15

**Authors:** Munveer Thind, David S. McKindley, James A. Reiffel, Gerald V. Naccarelli, John Stewart, Peter R. Kowey

**Affiliations:** ^1^ Division of Cardiology Lankenau Heart Institute Wynnewood Pennsylvania USA; ^2^ Sanofi Bridgewater New Jersey USA; ^3^ Division of Cardiology Columbia University New York City New York USA; ^4^ Division of Cardiology Penn State University College of Medicine Hershey Pennsylvania USA; ^5^ Sanofi Laval Quebec Canada

**Keywords:** antiarrhythmic drug, atrial fibrillation, atrial flutter, dronedarone, trough drug concentrations

## Abstract

**Background:**

In this post hoc analysis, we assessed patient characteristics as predictors of dronedarone trough concentrations and characterized the relationship of trough concentrations of dronedarone with its efficacy and safety.

**Hypothesis:**

Dronedarone is recommended as a 400 mg twice daily dose taken orally with meals. We hypothesize that drug concentration/bioavailability of dronedarone, measured as above‐ and below‐median trough concentrations, does not impact the efficacy outcomes.

**Methods:**

Average trough concentrations (C_trough_avg_) across multiple timepoints were calculated for each patient, and patient C_trough_avg_ values were categorized as below‐median or above‐median concentrations. The effect of patient baseline characteristics on dronedarone C_trough_avg_ was assessed in the below‐median versus above‐median groups. The effect of dronedarone in each C_trough_avg_ group versus placebo on risk of first atrial fibrillation/atrial flutter (AF/AFL) recurrence and safety was also evaluated.

**Results:**

Overall, 1795 plasma samples were available from 507 dronedarone‐treated patients. An above‐median C_trough_avg_ was associated with age ≥75 years, female sex, lower weight, higher pacemaker use, and higher oral anticoagulant use. The risk of adjudicated first AF/AFL recurrence was significantly lower with dronedarone versus placebo in the below‐median (hazard ratio [HR]: 0.71; 95% confidence interval [CI]: 0.56–0.91; *p* = .0054) and above‐median groups (HR: 0.63; 95% CI: 0.50–0.81; *p* = .0002). No difference in risk of AF/AFL recurrence was observed between the above‐ and below‐median groups. Safety and tolerability of dronedarone were similar between groups.

**Conclusion:**

Significant reduction in AF/AFL recurrence was observed in patients treated with dronedarone versus placebo, regardless of dronedarone concentrations above or below the median value.

## 1 INTRODUCTION

Atrial fibrillation (AF) is the most common sustained cardiac arrhythmia and is associated with increased risk of stroke, acute coronary syndrome, heart failure, and cardiovascular death.^1^, ^2^, ^3^, ^4^, ^5^ Guidelines for disease management suggest the use of antiarrhythmic drugs for the maintenance of sinus rhythm if a rhythm control strategy is warranted, depending on underlying heart disease and comorbidities.^1^, ^6^ Dronedarone is recommended for long‐term rhythm control in patients with no or minimal signs of structural heart disease, and in those with AF with normal or mildly impaired but stable left ventricular function (including heart failure with preserved ejection fraction), ischemic heart disease, or valvular heart disease.^6^ Dronedarone is indicated to reduce the risk of hospitalization for AF in patients in sinus rhythm with a history of paroxysmal or persistent AF.^7^

In the European Trial in Atrial Fibrillation or Flutter Patients Receiving Dronedarone for the Maintenance of Sinus Rhythm (EURIDIS; NCT00259428) and American–Australian–African Trial With Dronedarone in Atrial Fibrillation or Flutter Patients for the Maintenance of Sinus Rhythm (ADONIS; NCT00259376), dronedarone versus placebo significantly increased time to first documented AF/atrial flutter (AFL) recurrence and reduced ventricular rate during first recurrence in patients with nonpermanent AF/AFL.^8^ The pharmacokinetics (PKs) of dronedarone have been evaluated,^7^, ^9^, ^10^, ^11^ and the association of dronedarone dose with efficacy/safety has been reported;^12^ however, the association of dronedarone plasma concentrations with AF recurrence using the approved dose has not been previously published. Dronedarone is recommended as a 400 mg fixed dose taken orally twice daily (bid) with meals. In this post hoc analysis of the EURIDIS/ADONIS studies, we assessed patient characteristics as predictors of dronedarone trough concentrations and characterized the relationship of the trough concentrations of dronedarone with its efficacy and safety.

## 2 METHODS AND MATERIALS

### 2.1 Overview of the EURIDIS and ADONIS studies

EURIDIS and ADONIS were identically designed double‐blind, randomized, multicenter, Phase 3 studies conducted concurrently in which patients with nonpermanent AF/AFL were randomized to oral dronedarone 400 mg bid or placebo for 12 months.^8^ Patients who had experienced at least one episode of AF/AFL observed on electrocardiogram (ECG) in the preceding 3 months were eligible for enrollment. Patients were required to have been in sinus rhythm for ≥1 h to be eligible for randomization; baseline cardioversion to achieve sinus rhythm was permitted within 5 days before randomization. Additional study design and eligibility criteria details are published elsewhere.^8^ The primary endpoint of the EURIDIS/ADONIS studies was time to first documented recurrence of AF/AFL within 12 months; symptomatic AF/AFL recurrence and mean ventricular rate during first recurrence were also assessed. AF/AFL recurrence was defined as an episode lasting for ≥10 min, confirmed by two consecutive recordings taken 10 min apart on 12‐lead ECG or transtelephonic ECG monitoring. Safety assessments included adverse event (AE) reporting, vital signs, ECGs, and laboratory evaluations. AEs that occurred/worsened during study treatment or within 10 days after the last drug intake were categorized as treatment‐emergent adverse events (TEAEs).

### 2.2 Analysis of dronedarone trough concentrations

**Figure 1:**
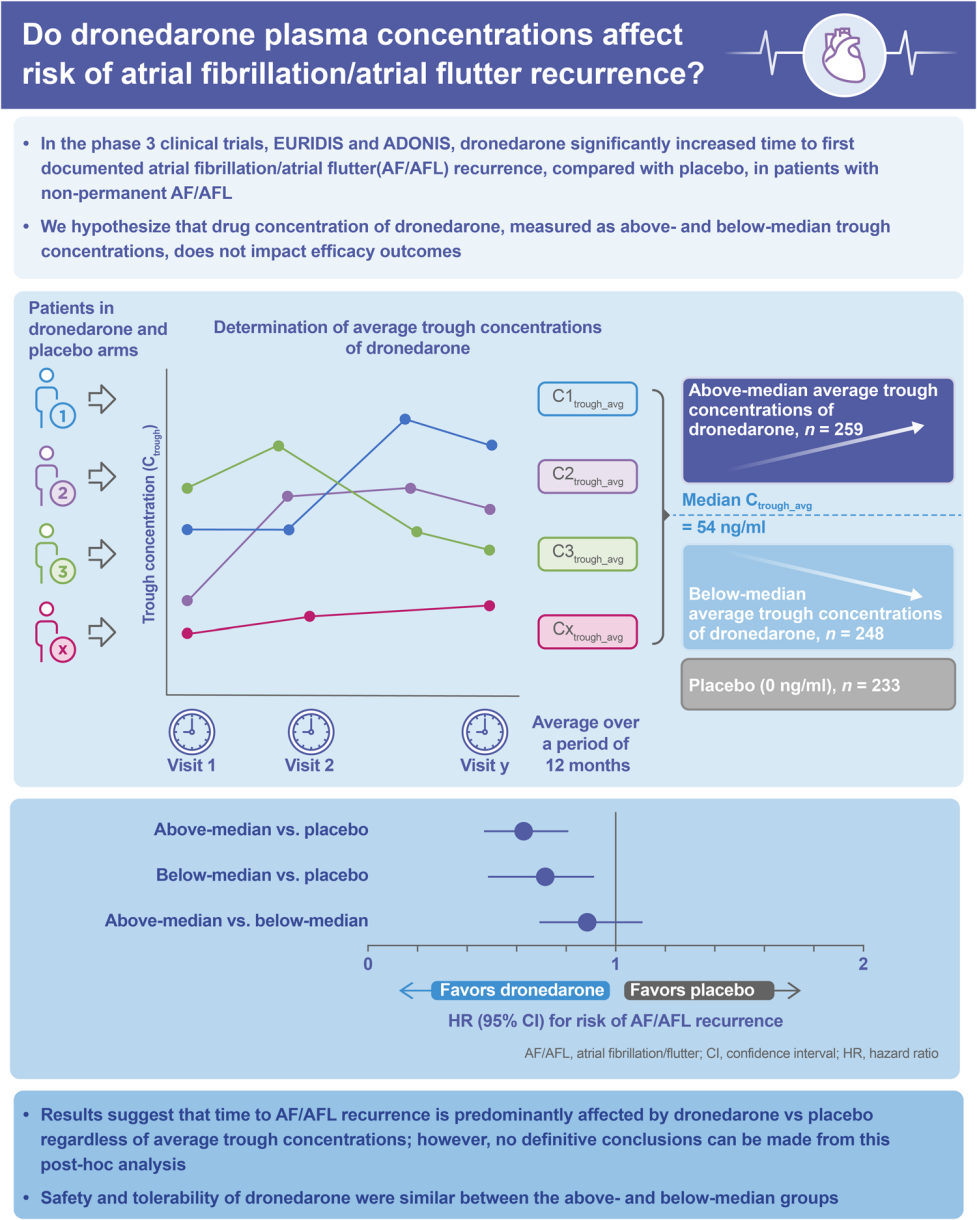
Study summary. C_trough_avg_, average trough concentration

In this pooled analysis, plasma concentrations for dronedarone were assessed for each patient in the dronedarone and placebo treatment arms on Days 7 ± 2, 21 ± 3, and Months 4 ± 5 days, 9 ± 5 days, and 12 ± 5 days. Samples were collected from a few patients on Day 14 ± 3, and Months 2 ± 5 days and 6 ± 5 days. All samples are assumed to be collected at steady state exposure, which is expected to be reached within 4–8 days of treatment, with peak dronedarone concentrations reached between 3 and 6 h after administration.^7^ Dronedarone concentrations were determined by a validated liquid chromatography with tandem mass spectrometry method with a quantification limit of 0.5 ng/ml. Concentrations were classified as at trough (C_trough_) if the time interval between the last dose of treatment and sampling was <2 h, or between 8 and 16 h. Average trough concentration (C_trough_avg_) over all timepoints was calculated for each patient as arithmetic mean. The median of these C_trough_avg_ values was determined, and patient C_trough_avg_ values were categorized as below‐median (<median) or above‐median (≥median) concentrations (Figure 1).

To identify factors affecting bioavailability of dronedarone, the effect of patient baseline characteristics on dronedarone C_trough_avg_ was assessed in the below‐median versus above‐median groups. The effect of dronedarone concentrations on risk of first AF/AFL recurrence was assessed in each C_trough_avg_ group versus placebo; effects on safety were also evaluated. As dronedarone can potentially affect ECG patterns and increase serum creatinine levels, it is important to understand how differences in C_trough_avg_ values may affect this. To determine any change in ECG patterns and serum creatinine levels from baseline (baseline patients are in sinus rhythm), a median on‐study value from all assessments (Days 7 and 21, and Months 4, 9, and 12) was calculated for each patient, and used to calculate an overall median on‐study value for all patients within each C_trough_avg_ group for comparison with the baseline value.

The effect of demographic and disease characteristics at baseline and change in ECG parameters (among patients in sinus rhythm) after treatment on time to AF/AFL recurrence in the dronedarone C_trough_avg_ below‐median, dronedarone C_trough_avg_ above‐median, and placebo groups was also assessed.

### 2.3 Statistical analysis

Baseline characteristics by dronedarone C_trough_avg_ and placebo groups are reported descriptively. To assess the effect of dronedarone in each C_trough_avg_ group versus the placebo group on time to first AF/AFL recurrence, cumulative incidence functions for each group were calculated with the Prentice nonparametric estimator. To identify baseline characteristics or change from baseline in ECG parameters that had an effect on the risk of first AF/AFL, a Cox regression analysis was used to compare each C_trough_avg_ group versus placebo, and to compare the below‐median and above‐median dronedarone C_trough_avg_ groups. *p* values, calculated using the logrank test, were not adjusted due to the post hoc nature of this analysis. Data were analyzed with SAS version 9.4 (SAS Institute).

## 3 RESULTS

**Figure 2:**
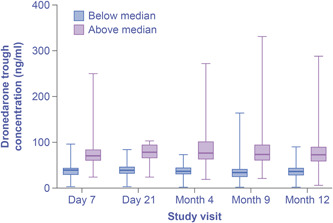
Dronedarone C_trough_ concentrations by study visit. Although included in the analysis, data collected at visits on Day 14 and Months 2 and 6 were limited. Only timepoints with *n* > 10 have been included on the chart. Boxes indicate Q1, median, and Q3. Range indicated by lines

In this analysis, 1795 plasma samples were available from 507 patients treated with dronedarone; 760 samples were available from 233 patients treated with placebo in the EURIDIS and ADONIS trials. Placebo samples were included as negative controls. Dronedarone C_trough_ concentrations by study visit are shown in Figure 2. The median dronedarone C_trough_avg_ value was 54 ng/ml; 248 patients had C_trough_avg_ values <54 ng/ml and 259 patients had C_trough_avg_ values ≥54 ng/ml. Dronedarone was not detected in patients receiving placebo. The majority of samples were obtained during scheduled visits for assessment of the primary endpoint (Days 7 and 21, and Months 4, 9, and 12), so the PK data align with the sampling points for ECG and serum creatinine (Figure 2).

The overall study findings are summarized in Figure 1.

### 3.1 Patient baseline characteristics by dronedarone C_trough_avg_

Baseline characteristics are reported in Table 1. The above‐median versus below‐median group had more patients ≥75 years of age (21.2% vs. 6.5%), more females (44.8% vs. 16.5%), lower body weight (mean ± standard deviation [SD]: 79.8 kg ± 14.7 vs. 93.3 kg ± 18.4), and lower body mass index (mean ± SD: 27.9 ± 4.8 vs. 30.1 ± 5.8). A CHA_2_DS_2_‐VASc score of ≥2 was observed in 70.3% of patients in the above‐median group, and in 47.6% of patients in the below‐median group. A higher percentage of patients in the above‐median versus below‐median group had implanted pacemakers (10.4% vs. 2.8%) and used oral anticoagulants (71.6% vs. 59.1%). The proportions of patients who received moderate inhibitors of CYP3A4 were similar in the below‐median (18.1%) and above‐median (16.7%) groups.

**Table 1:** Demographic and disease characteristics at baseline

Parameter	Dronedarone C_trough_avg_ below‐median^a^ (*n* = 248)	Dronedarone C_trough_avg_ above‐median^a^ (*n* = 259)	Placebo (*n* = 233)
Age, years			
Mean (SD)	60.1 (11.4)	66.2 (10.1)	63.2 (11.4)
Median (Q1, Q3)	61.0 (53.0, 69.0)	66.0 (60.0, 74.0)	66.0 (55.0, 71.0)
<65 years, *n* (%)	145 (58.5)	107 (41.3)	110 (47.2)
65 to <75 years, *n* (%)	87 (35.1)	97 (37.5)	91 (39.1)
≥75 years, *n* (%)	16 (6.5)	55 (21.2)	32 (13.7)
Sex, *n* (%)			
Male	207 (83.5)	143 (55.2)	158 (67.8)
Female	41 (16.5)	116 (44.8)	75 (32.2)
Weight, kg			
Mean (SD)	93.3 (18.4)^b^	79.8 (14.7)^c^	87.3 (17.5)^c^
Median (Q1, Q3)	90.5 (82.7, 101.5)^b^	79.0 (70.0, 89.0)^c^	85.0 (75.0, 95.5)^c^
BMI, kg/m^2^			
Mean (SD)	30.1 (5.8)^d^	27.9 (4.8)^e^	28.9 (4.7)^f^
Median (Q1, Q3)	28.7 (26.5, 32.4)^d^	27.3 (24.7, 30.5)^e^	28.3 (26.1, 31.0)^f^
Serum creatinine, mg/dl (µmol/L)			
Mean	1.1 (100)	1.1 (97)	1.1 (97)
SD	0.2 (17)	0.2 (18)^c^	0.2 (18)^b^
eGFR category (CKD‐EPI formula, *n* (%), ml/min			
<30	0	0^c^	2 (0.9)^b^
30–44	7 (2.8)	24 (9.3)^c^	12 (5.2)^b^
45–59	60 (24.2)	91 (35.4)^c^	58 (25.0)^b^
60–89	154 (62.1)	129 (50.2)^c^	140 (60.3)^b^
≥90	27 (10.9)	13 (5.1)^c^	20 (8.6)^b^
CHA_2_DS_2_‐VASc score, *n* (%)			
0–1	130 (52.4)	77 (29.7)	96 (41.2)
2–3	97 (39.1)	127 (49.0)	103 (44.2)
>3	21 (8.5)	55 (21.2)	34 (14.6)
Baseline cardiovascular examination			
2D left ventricular ejection fraction <35%, *n* (%)	7 (2.9)^g^	8 (3.2)^i^	8 (3.6)^i^
Left chronic heart failure (NYHA Class ≥1), *n* (%)	28 (11.3)	48 (18.5)	39 (16.7)
Left atrium diameter (mm), mean (SD)	42.5 (7.1)^e^	42.4 (7.3)^f^	42.9 (6.7)^h^
Cardiovascular history, *n* (%)			
Structural heart disease	105 (42.7)^c^	117 (45.3)^b^	95 (41.7)^e^
Coronary heart disease	60 (24.2)	60 (23.2)	49 (21.0)
Dilated cardiomyopathy	12 (4.8)	18 (6.9)	18 (7.7)
Hypertension	142 (57.3)	164 (63.3)	120 (51.5)
Valvular heart disease	30 (12.1)	51 (19.7)	36 (15.5)
Hypertrophic cardiomyopathy	6 (2.4)	9 (3.5)	7 (3.0)
Congenital heart disease	2 (0.8)	8 (3.1)	1 (0.4)
Implanted cardioverter defibrillator	2 (0.8)	1 (0.4)	4 (1.7)
Pacemaker	7 (2.8)	27 (10.4)	11 (4.7)
Rheumatic heart disease	7 (2.8)	10 (3.9)	9 (3.9)
Concomitant medications, *n* (%)			
Beta blockers (except sotalol)	135 (54.4)	140 (54.1)	144 (61.8)
ACE/angiotensin II inhibitor	120 (51.7)^j^	135 (52.5)^c^	117 (51.3)^e^
Digoxin	39 (15.7)	39 (15.1)	45 (19.3)
Calcium antagonists with heart rate lowering effects	41 (17.7)^j^	42 (16.3)^c^	40 (17.5)^e^
Oral anticoagulants	137 (59.1)^j^	184 (71.6)^c^	158 (69.3)^e^
Statins metabolized by CYP3A4	53 (22.8)^j^	57 (22.2)^c^	59 (25.9)^e^
Statins not metabolized by CYP3A4	29 (12.5)^j^	42 (16.3)^c^	35 (15.4)^e^
Moderate inhibitors of CYP3A4	42 (18.1)^j^	43 (16.7)^c^	42 (18.4)^e^

Abbreviations: 2D, two dimensional; ACE, angiotensin‐converting enzyme; BMI, body mass index; CKI‐EPI, Chronic Kidney Disease Epidemiology Collaboration; C_trough_avg_, average trough concentration; eGFR, estimated glomerular filtration rate; NYHA, New York Heart Association; SD, standard deviation.

^a^ Median dronedarone C_trough_avg_ = 54 ng/ml; below‐median is <54 ng/ml and above‐median is ≥54 ng/ml.

^b^ Data were missing for one patient.

^c^ Data were missing for two patients.

^d^ Data were missing for three patients.

^e^ Data were missing for five patients.

^f^ Data were missing for six patients

^g^ Data were missing for nine patients.

^h^ Data were missing for 10 patients.

^i^ Data were missing for 11 patients.

^j^ Data were missing for 16 patients.

### 3.2 Risk of first AF/AFL recurrence within 12 months by dronedarone C_trough_avg_

**Figure 3:**
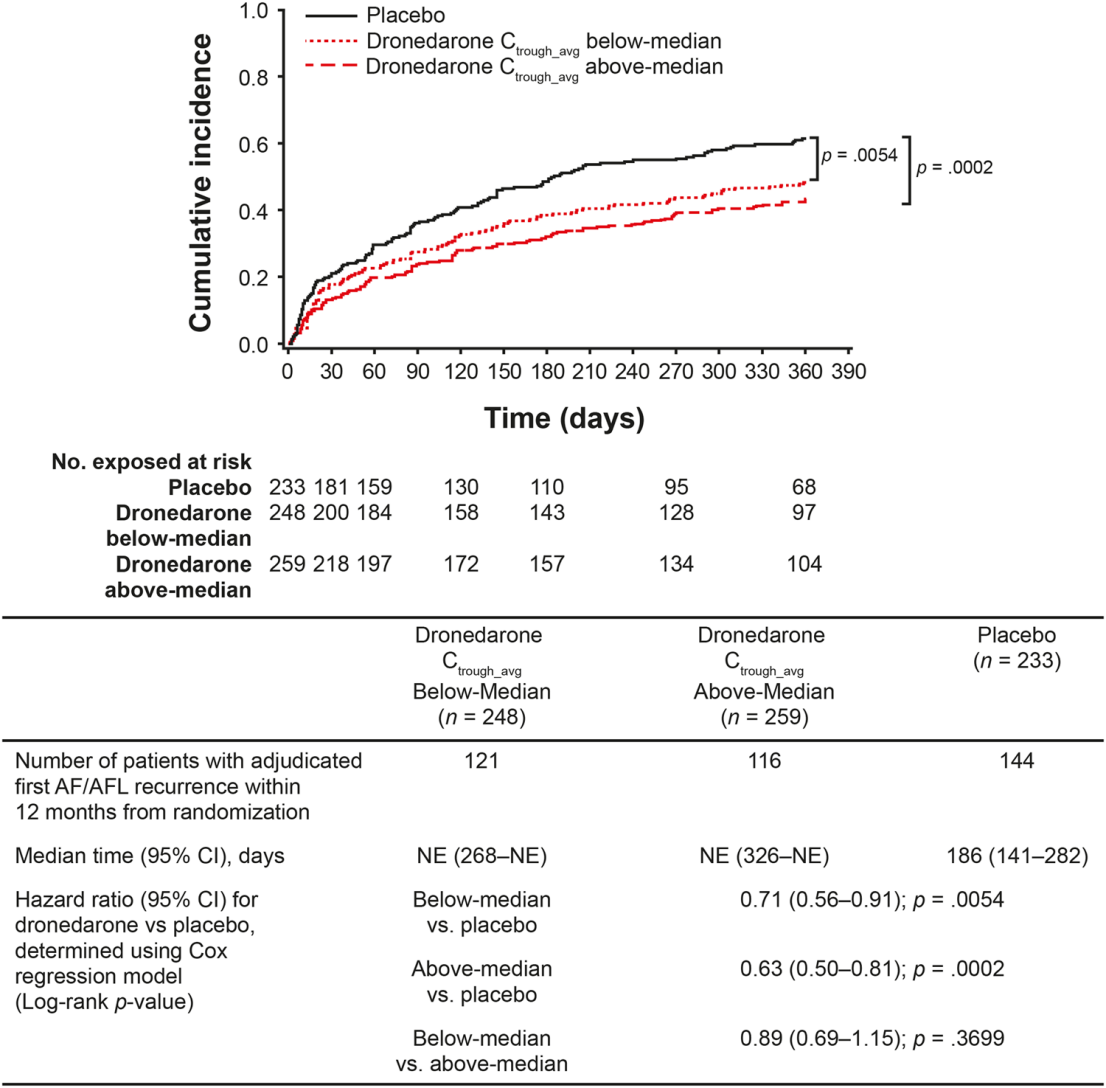
Time to adjudicated first AF/AFL recurrence among patients with below‐median^a^ or above‐median^a^ values of C_trough_avg_ of dronedarone versus placebo. ^a^Median dronedarone C_trough_avg_ = 54 ng/ml; below‐median is <54 ng/ml and above‐median is ≥54 ng/ml. AF/AFL, atrial fibrillation/atrial flutter; CI, confidence interval; C_trough_avg_, average trough concentration; NE, not estimated

The risk of adjudicated first AF/AFL recurrence was significantly lower with dronedarone versus placebo in both the below‐median (hazard ratio [HR]: 0.71; 95% confidence interval [CI]: 0.56–0.91; *p* = .0054) and above‐median groups (HR: 0.63; 95% CI: 0.50–0.81; *p* = .0002). No significant difference was observed between the above‐median versus below‐median groups (HR: 0.89; 95% CI: 0.69–1.15; *p* = .3699) (Figure 3). Similar results were observed for risk of first symptomatic AF/AFL recurrence with HR of 0.78 (95% CI: 0.60–1.01; *p* = .0613) for the below‐median group versus placebo, 0.61 (95% CI: 0.47–0.80; *p* = .0003) for the above‐median group versus placebo, and 0.78 (95% CI: 0.59–1.04; *p* = .0861) for the above‐median group versus the below‐median group.

### 3.3 Variables affecting risk of first AF/AFL recurrence

Cox analyses showed that when compared with placebo, dronedarone C_trough_avg_ below‐median and above‐median groups had a reduced risk of first AF/AFL for most patient subgroups at baseline or change in ECG characteristics after treatment (Figure S1). HRs by subgroups of patient characteristics for below‐median versus above‐median dronedarone C_trough_avg_ groups were comparable.

### 3.4 C_trough_avg_ values of dronedarone and safety

TEAEs were observed in 70.7% and 75.0% of patients in the above‐median and below‐median dronedarone groups, respectively, and 70.8% of those receiving the placebo. AF (6.9% and 8.1%, respectively) and diarrhea (8.1% and 7.7%, respectively) were the most common TEAEs in both the above‐median and below‐median dronedarone groups (Table 2). TEAEs leading to discontinuation were more common in the above‐median group (11.2%) compared with the below‐median group (3.2%), and placebo (6.4%), with a higher proportion of patients discontinuing in the dronedarone groups due to nausea (1.2% vs. 0%), increased blood creatinine (1.5% vs. 0%), headache (1.5% vs. 0%), and dyspnea (1.2% vs. 0.8%) (Table S1). In the placebo group, 1.3% of patients discontinued due to AF, versus 0% in both dronedarone groups. The rates of serious TEAEs were similar in the above‐median (22.4%) and below‐median groups (18.1%), and in those receiving placebo (24.9%) (Table 2 and Table S2). Deaths due to any cause were reported in one patient (0.4%) in the below‐median group and three patients (1.2%) in the above‐median group. ECG parameters and on serum creatinine concentrations by dronedarone C_trough_avg_ are reported in Tables S3 and S4, respectively.

**Table 2:** Treatment‐emergent adverse events

Event	Dronedarone C_trough_avg_ below‐median^a^ (*n* = 248)	Dronedarone C_trough_avg_ above‐median^a^ (*n* = 259)	Placebo (*n* = 233)
Summary of treatment‐emergent adverse events			
Patients with ≥1 event	186 (75.0)	183 (70.7)	165 (70.8)
Patients with ≥1 event leading to treatment discontinuation	8 (3.2)	29 (11.2)	15 (6.4)
Patients with ≥1 serious event	45 (18.1)	58 (22.4)	58 (24.9)
Patients who died	1 (0.4)	3 (1.2)	0
Treatment‐emergent adverse events reported in ≥3% of patients in any group			
Cardiac disorders			
Atrial fibrillation	20 (8.1)	18 (6.9)	21 (9.0)
Bradycardia	9 (3.6)	4 (1.5)	3 (1.3)
Angina pectoris	3 (1.2)	9 (3.5)	8 (3.4)
Gastrointestinal disorders			
Diarrhea	19 (7.7)	21 (8.1)	12 (5.2)
Nausea	9 (3.6)	12 (4.6)	9 (3.9)
Dyspepsia	4 (1.6)	4 (1.5)	7 (3.0)
Upper abdominal pain	2 (0.8)	5 (1.9)	7 (3.0)
General disorders			
Peripheral edema	11 (4.4)	13 (5.0)	15 (6.4)
Fatigue	5 (2.0)	10 (3.9)	8 (3.4)
Infections			
Influenza	10 (4.0)	4 (1.5)	11 (4.7)
Nasopharyngitis	8 (3.2)	4 (1.5)	5 (2.1)
Bronchitis	6 (2.4)	8 (3.1)	2 (0.9)
Upper respiratory tract infection	11 (4.4)	8 (3.1)	4 (1.7)
Urinary tract infection	2 (0.8)	10 (3.9)	2 (0.9)
Investigations			
Increased blood creatinine	5 (2.0)	10 (3.9)	1 (0.4)
Increased blood creatinine phosphokinase	9 (3.6)	3 (1.2)	1 (0.4)
Musculoskeletal and connective tissue disorders			
Back pain	13 (5.2)	8 (3.1)	8 (3.4)
Arthralgia	10 (4.0)	6 (2.3)	4 (1.7)
Pain in extremity	3 (1.2)	8 (3.1)	3 (1.3)
Nervous system disorders			
Headache	10 (4.0)	17 (6.6)	24 (10.3)
Respiratory, thoracic, and mediastinal disorders			
Dyspnea	7 (2.8)	9 (3.5)	8 (3.4)
Cough	5 (2.0)	9 (3.5)	6 (2.6)
Vascular disorders			
Hypertension	9 (3.6)	7 (2.7)	9 (3.9)

Abbreviation: C_trough_avg_, average trough concentration.

^a^ Median dronedarone C_trough_avg_ = 54 ng/ml; below‐median is <54 ng/ml and above‐median is ≥54 ng/ml.

## 4 DISCUSSION

This post hoc analysis of the EURIDIS and ADONIS studies demonstrated that dronedarone significantly reduced the risk of adjudicated first AF/AFL recurrence among patients with below‐median as well as above‐median C_trough_avg_ compared with the placebo group. A similar result was observed for symptomatic first AF/AFL recurrence where there was a significant difference only in the above‐median versus placebo groups. In this analysis, there was no significant difference in the risk of adjudicated or symptomatic AF/AFL recurrence among patients in the two dronedarone groups.

In the primary analysis of the EURIDIS/ADONIS studies, median time to AF/AFL recurrence of 116 days with dronedarone and 53 days for placebo was reported.^8^ In the current analysis, time to AF/AFL recurrence with dronedarone was associated with event rates <50% in the above‐median or below‐median groups; therefore, estimated values for median times could not be calculated for the dronedarone subgroups. Median time to AF/AFL recurrence for the placebo group was calculated to be 186 days. However, we were able to successfully recreate the Kaplan–Meier plots for the primary analysis; it is likely that the differences are due to smaller subpopulation and reduced event rates in the current analyses.

The Dose Adjustment For Normal Eating clinical study investigated time to first AF recurrence in patients with long‐standing AF using dronedarone bid doses of 400, 600, and 800 mg. The study did not demonstrate any significant improvement in time to first recurrence of AF compared with the 400 mg bid dose.^12^ The 400 mg dose provided an acceptable balance between efficacy and safety, as gastrointestinal side effects seemed to be dose‐related in the study. To increase dronedarone exposure, administration with a meal is recommended (4% without food, ∼15% with a high‐fat meal).^7^ The results of this post hoc analysis support the use of dronedarone at the indicated dose of 400 mg bid with meals.

At baseline, age ≥75 years, female sex, lower weight, higher pacemaker use, and higher oral anticoagulant use were associated with above‐median C_trough_avg_. Dronedarone exposure is known to be 23% higher in patients ≥65 years old compared with those <65 years of age,^7^ reflected by the higher proportion of older patients observed in the above‐median C_trough_avg_ group in the current study. This observation is likely due to changes in hepatic function, increased comorbidities, and the increased risk of drug–drug interactions due to the increased use of concomitant medications expected with increasing age.^13^ The greater C_trough_avg_ concentrations observed in the female population are consistent with the known PK properties of dronedarone and are likely linked to BMI, as patients with a body weight of 60 kg have a 1.4‐fold greater plasma exposure than in those with a body weight of 60–100 kg.^14^ The distribution of CHAD_2_S_2_‐VASc scores was consistent with that of age and sex, with higher scores observed in a greater proportion of patients in the above‐median versus below‐median C_trough_avg_ groups.

The efficacy of dronedarone, as measured by time to first AF/AFL recurrence, versus the placebo, was maintained across patient subgroups for both the above‐ and below‐median C_trough_avg_ groups. When comparing the above‐ and below‐median C_trough_avg_ groups, efficacy was consistent across patient subgroups. These results suggest that time to AF/AFL recurrence is predominantly affected by dronedarone versus placebo regardless of average trough concentrations. However, no definitive conclusions could be drawn owing to the wide CIs resulting from small sample sizes as well as small numbers of events.

As dronedarone is extensively metabolized by CYP3A4,^15^ the effect of coadministration of moderate CYP3A4 inhibitors was included in the Cox regression analysis assessing the effect on time to AF/AFL recurrence. Moderate inhibitors of CYP3A4, such as the calcium channel blockers verapamil and diltiazem, are often coprescribed with dronedarone, and have been shown to lead to a 1.5‐ and 1.7‐fold increase in maximum concentration and area under the curve value of dronedarone at standard doses, respectively.^11^, ^16^ However, in this study, the distributions of patients with plasma concentrations of dronedarone below‐median and above‐median were similar in patients using moderate CYP3A4 inhibitors. As the relative timing of the coadministration of the moderate CYP3A inhibitors and dronedarone was not known and adherence to intake of the CYP3A4 inhibitors was not tracked, it is hard to draw any conclusions from these data.

The safety of dronedarone was comparable in the below‐median and above‐median groups. Consistent with the EURIDIS/ADONIS results, dronedarone treatment in the above‐ and below‐median groups was associated with longer QT, Bazett‐corrected QT, and PR intervals, and lower heart rate compared with baseline and placebo.^8^, ^15^ The increase in QT interval is small, a known Class III effect, and consistent with the mechanism of action of dronedarone.^17^ However, dronedarone treatment is not associated with an increased risk of ventricular proarrhythmias such as torsade de pointes,^8^, ^12^, ^18^, ^19^, ^20^ likely due to blocking of sodium (Class I activity) and calcium channels (Class IV activity).^7^, ^21^, ^22^, ^23^, ^24^, ^25^

Serum creatinine concentrations were observed to increase from baseline in both the above‐ and below‐median groups (mean increase [SD]: 0.11 [0.14] mg/dl [9 [12] µmol/l] and 0.08 [0] mg/dl [7 [10] µmol/l], respectively) whereas the placebo group maintained similar levels of serum creatinine on‐study from baseline (mean change [SD]: −0.01 [0.11] mg/dl [−1 [10] µmol/l]). Dronedarone treatment is known to result in an increase in serum creatinine concentrations because of partial inhibition of tubular transport of creatinine.^11^ However, dronedarone, which is minimally eliminated via the renal route (approximately 6%), has no effect on glomerular filtration rate, so no dose adjustments for people with renal impairment are required.^7^

### 4.1 Limitations

There are limitations of this analysis that should be considered. Not all patients had trough levels measured. As a relatively low number of trough concentrations were available, we were unable to analyze each patient's trough concentration versus time to first recurrence using this type of methodology. Therefore, each patient's average trough concentration from throughout the study was used to calculate the median values that form the C_trough_ categories. As C_trough_avg_ was used to correlate to time to first recurrence, it was not possible to assess potential lag time that may be associated with the pharmacodynamic response (e.g., AF recurrence or QT prolongation) to dronedarone. In addition, concomitant drugs and timing of dronedarone administration relative to meals were not controlled throughout the study; meals were also not standardized. These are potential variables that can alter dronedarone trough concentrations and add variability into the data that cannot be controlled. Multiple small patient subcategories resulted in a reduced power to detect significant changes in the risk analysis; hence, data on subgroups should be considered only exploratory. Finally, it should be noted that this sample was nonrandomized and may not be representative of the total population of the EURIDIS/ADONIS studies.

## 5 CONCLUSION

Dronedarone plasma concentrations from a large clinical trial have not been previously published, so these results add to the existing understanding of dronedarone administration. Dronedarone in the above‐median and below‐median C_trough_avg_ groups was associated with a lower risk of AF/AFL recurrence compared with placebo. No difference in risk of AF/AFL recurrence was observed between the above‐ and below‐median groups (supporting the use of the current 400 mg bid dose) and across populations in which the trough concentrations may vary. An above‐median C_trough_avg_ was associated with age ≥75 years, female sex, lower weight, higher pacemaker use, and higher oral anticoagulant use; the higher CHAD_2_S_2_‐VASc score observed in the above‐median C_trough_avg_ group was consistent with the higher age and higher proportion of female sex observed in that group. The safety and tolerability of dronedarone were similar between the above‐ and below‐median groups.

## CONFLICT OF INTERESTS

David S. McKindley and John Stewart are employees of Sanofi US Inc. James A. Reiffel has received personal fees (during the study) from Sanofi and personal fees (outside of the submitted work) from Acesion Pharma, Medtronic, Janssen Pharmaceuticals, and Roivant Sciences. Gerald V. Naccarelli has received the following (outside of the submitted work): personal fees from Acesion Pharma, GlaxoSmithKline, Janssen Pharmaceuticals, Milestone Pharmaceuticals, Omeicos Therapeutics, and Sanofi and grants from Janssen Pharmaceuticals. Peter R. Kowey is an ad hoc consultant for Sanofi. Munveer Thind has no conflict of interests.

## Supporting information

Supplementary information.

## Data Availability

Qualified researchers may request access to patient‐level data and related study documents including the clinical study report, study protocol with any amendments, blank case report form, statistical analysis plan, and dataset specifications. Patient level data will be anonymized and study documents will be redacted to protect the privacy of our trial participants. Further details on Sanofi's data‐sharing criteria, eligible studies, and process for requesting access can be found at https://www.clinicalstudydatarequest.com/
