# Supplementary material for: Predictors of dronedarone plasma drug concentrations and effect on atrial fibrillation/atrial flutter recurrence: Analyses from the EURIDIS and ADONIS studies
Source: Clin Cardiol. 2022 Jan 15;45(1):119–28. doi: 10.1002/clc.23768 (PMC8799059; doi:10.1002/clc.23768)
Supplement: Supplementary file 1 — Supplementary information. [file CLC-45-119-s001.docx]

**Supplementary Materials**

**Supplemental Table S1.** Treatment-emergent adverse events leading to treatment discontinuation.

| Event, *n* (%) | Dronedarone C_trough_avg_  Below-Median^a^  (*n* = 248) | Dronedarone C_trough_avg_  Above-Median^a^  (*n* = 259) | Placebo  (*n* = 233) |
| --- | --- | --- | --- |
| Patients with ≥1 treatment-emergent adverse event leading to treatment discontinuation | 8 (3.2) | 29 (11.2) | 15 (6.4) |
| Treatment-emergent adverse events leading to treatment discontinuation in ≥1% patients in any group | | | |
| Cardiac disorders |  |  |  |
| Atrial fibrillation | 0 | 0 | 3 (1.3) |
| Gastrointestinal disorders |  |  |  |
| Diarrhea | 1 (0.4) | 1 (0.4) | 3 (1.3) |
| Nausea | 0 | 3 (1.2) | 1 (0.4) |
| Investigations |  |  |  |
| Increased blood creatinine | 0 | 4 (1.5) | 0 |
| Nervous system disorders |  |  |  |
| Headache | 0 | 4 (1.5) | 1 (0.4) |
| Respiratory, thoracic, and mediastinal disorders |  |  |  |
| Dyspnea | 2 (0.8) | 3 (1.2) | 0 |
| Abbreviation: C_trough_avg_, average trough concentration.  ^a^ Median dronedarone C­_trough_avg_ = 54 ng/ml; below-median is <54 ng/ml and above-median is ≥54 ng/ml. | | | |

**Supplemental Table S2.** Serious treatment-emergent adverse events.

| Event, *n* (%) | Dronedarone C_trough_avg_  Below-Median^a^  (*n* = 248) | Dronedarone C_trough_avg_  Above-Median^a^  (*n* = 259) | Placebo  (*n* = 233) |
| --- | --- | --- | --- |
| Patients with ≥1 serious treatment-emergent adverse event | 45 (18.1) | 58 (22.4) | 58 (24.9) |
| Serious treatment-emergent adverse events reported in ≥1% patients in any group | | | |
| Cardiac disorders |  |  |  |
| Atrial fibrillation | 20 (8.1) | 18 (6.9) | 21 (9.0) |
| Angina pectoris | 1 (0.4) | 4 (1.5) | 4 (1.7) |
| Cardiac failure | 1 (0.4) | 3 (1.2) | 0 |
| Atrial flutter | 0 | 0 | 4 (1.7) |
| General disorders |  |  |  |
| Chest pain | 3 (1.2) | 0 | 1 (0.4) |
| Infections |  |  |  |
| Urinary tract infection | 0 | 3 (1.2) | 1 (0.4) |
| Abbreviation: C_trough_avg_, average trough concentration.  ^a^ Median dronedarone C­_trough_avg_ = 54 ng/ml; below-median is <54 ng/ml and above-median is ≥54 ng/ml. | | | |

**Supplemental Table S3.** Electrocardiogram characteristics^a^ at baseline and on-study. ECG characteristics were similar between groups. Among patients in sinus rhythm, lower heart rate, longer PR interval, longer QT interval, and a longer Bazett-corrected QT interval were observed on-study for both the above- and below-median groups compared with placebo. Lower heart rate, longer PR interval, longer QT interval, and a longer Bazett-corrected QT interval were additionally observed for both the below- and above-median groups, compared with baseline levels. On-study ECG characteristics were similar to baseline for the placebo group.

|  | Baseline | | | On-study | | |
| --- | --- | --- | --- | --- | --- | --- |
| Parameter | **Dronedarone C_trough_avg_  below-median^b^**  **(*n* = 248)** | **Dronedarone C_trough_avg_  above-median^b^**  **(*n* = 259)** | **Placebo**  ***(n* = 233)** | **Dronedarone C_trough_avg_  below-median^b^**  **(*n* = 248)** | **Dronedarone C_trough_avg_  above-median^b^**  **(*n* = 259)** | **Placebo**  **(*n* = 233)** |
| Heart rate, bpm |  |  |  |  |  |  |
| *n* | 242 | 248 | 224 | 242 | 242 | 223 |
| Mean (SD) | 64.6 (10.8) | 64.5 (10.0) | 63.3 (10.2) | 61.0 (9.3) | 60.7 (8.2) | 63.9 (9.3) |
| Median (Q1, Q3) | 63.0 (56.0, 71.0) | 63.0 (57.0, 70.0) | 61.5 (56.0, 69.0) | 60.0 (54.0, 67.0) | 59.3 (55.0, 65.0) | 63.0 (57.5, 69.0) |
| PR interval, ms |  |  |  |  |  |  |
| *n* | 241 | 245 | 223 | 241 | 239 | 222 |
| Mean (SD) | 172.9 (29.7) | 169 (29.6) | 173.7 (31.0) | 176.4 (27.4) | 176.4 (25.8) | 170.8 (26.6) |
| Median (Q1, Q3) | 171.0 (153.0, 187.0) | 167.0 (148.0, 188.0) | 168.0 (152.0, 189.0) | 172.0 (158.0, 188.5) | 175.0 (159.5, 192.0) | 166.5 (152.5, 183.0) |
| QRS interval, ms |  |  |  |  |  |  |
| *n* | 242 | 246 | 224 | 242 | 240 | 223 |
| Mean (SD) | 90.6 (15.1) | 93.6 (20.2) | 91.4 (17.6) | 91.5 (13.1) | 94.0 (19.5) | 91.3 (14.7) |
| Median (Q1, Q3) | 90.0 (82.0, 96.0) | 90.0 (82.0, 96.0) | 90.0 (81.0, 96.0) | 90.0 (85.5, 95.0) | 90.0 (84.0, 95.0) | 88.5 (84.5, 93.0) |
| QT interval, ms |  |  |  |  |  |  |
| *n* | 238 | 239 | 212 | 237 | 233 | 209 |
| Mean (SD) | 393.7 (39.5) | 407.9 (39.0) | 403.4 (42.1) | 409.8 (32.2) | 427.1 (34.4) | 394.7 (30.8) |
| Median (Q1, Q3) | 392.5 (365.0, 416.0) | 406.0 (379.0, 433.0) | 401.0 (373.0, 430.0) | 410.0 (388.0, 431.0) | 425.0 (404.5, 444.5) | 393.0 (372.5, 413.0) |
| QTc Bazett, ms |  |  |  |  |  |  |
| *n* | 238 | 239 | 212 | 237 | 233 | 209 |
| Mean (SD) | 405.4 (34.2) | 419.5 (33.6) | 411.6 (36.7) | 411.2 (25.6) | 427.8 (28.4) | 405.0 (26.7) |
| Median (Q1, Q3) | 401.5 (382.0, 428.0) | 417.0 (395.0, 411.0) | 405.5 (385.0, 433.5) | 411.0 (392.0, 427.0) | 426.0 (407.5, 441.0) | 404.0 (386.0, 425.0) |
| Abbreviations: C_trough_avg_, average trough concentration; ECG, electrocardiogram; SD, standard deviation.  ^a^ Only 12-lead ECGs in normal sinus rhythm were considered.  ^b^ Median dronedarone C­_trough_avg_ = 54 ng/ml; below-median is <54 ng/ml and above-median is ≥54 ng/ml. | | | | | | |

**Supplemental Table S4.** Serum creatinine levels at baseline and on-study. Baseline serum creatinine levels were similar between groups. On-study mean (SD) serum creatinine concentrations were higher in both the above- and below-median groups compared with placebo (1.21 [0.23] and 1.21 [0.20] vs. 1.10 [0.18] mg/dL;107 [21] and 107[18] vs. 96 [16] µmol/l, respectively). Compared with baseline, on-study serum creatinine concentrations were similar in the placebo group (mean change of: −0.01 [0.11] mg/dL; −1 [8] µmol/l) and increased by a mean of 0.08 (0.11) md/dL (7 [10] µmol/l) and 0.11 (0.14) mg/dL ((9 [12] µmol/l) in the below-median and above-median groups, respectively.

| Parameter | Dronedarone C_trough_avg_  below-median^a^  (*n* = 248) | Dronedarone C_trough_avg_  above-median^a^  (*n* = 259) | Placebo  (*n* = 233) |
| --- | --- | --- | --- |
| Baseline serum creatinine, mg/dl (µmol/l) | |  |  |
| *n* | 248 | 257 | 232 |
| Mean  SD | 1.13 (100)  0.19 (17) | 1.10 (97)  0.21 (18) | 1.10 (97)  0.20 (18) |
| Median  Q1  Q3 | 1.11 (98)  1.01 (89)  1.25 (111) | 1.09 (96)  0.94 (83)  1.21 (107) | 1.10 (97)  0.97 (86)  1.23 (109) |
| On-study serum creatinine, mg/dl (µmol/l) | |  |  |
| *n* | 248 | 259 | 233 |
| Mean  SD | 1.21 (107)  0.20 (18) | 1.21 (107)  0.23 (21) | 1.09 (96)  0.18 (16) |
| Median  Q1  Q3 | 1.19 (105)  1.07 (95)  1.33 (118) | 1.19 (105)  1.06 (94)  1.31 (116) | 1.10 (97)  0.97 (86)  1.19 (105) |
| Change from baseline in serum creatinine, mg/dl (µmol/l) | |  |  |
| *n* | 248 | 257 | 232 |
| Mean  (SD) | 0.08 (7)  0.11 (10) | 0.11 (9)  0.14 (12) | −0.01 (−1)  0.11 (10) |
| Median  Q1  Q3 | 0.08 (7)  0.01 (1)  0.14 (12) | 0.10 (9)  0.02 (2)  0.18 (16) | −0.01 (−1)  −0.08 (−7)  0.04 (4) |
| Abbreviations: C_trough_avg_, average trough concentration; SD, standard deviation.  Concentrations converted from umol/l, as originally reported.  ^a^ Median dronedarone C­_trough_avg_ = 54 ng/ml; below-median is <54 ng/ml and above-median is ≥54 ng/ml. | | | |

**Supplementary Figure S1.** Forest plot of hazard ratio of adjudicated first AF/AFL recurrence within 12 months.


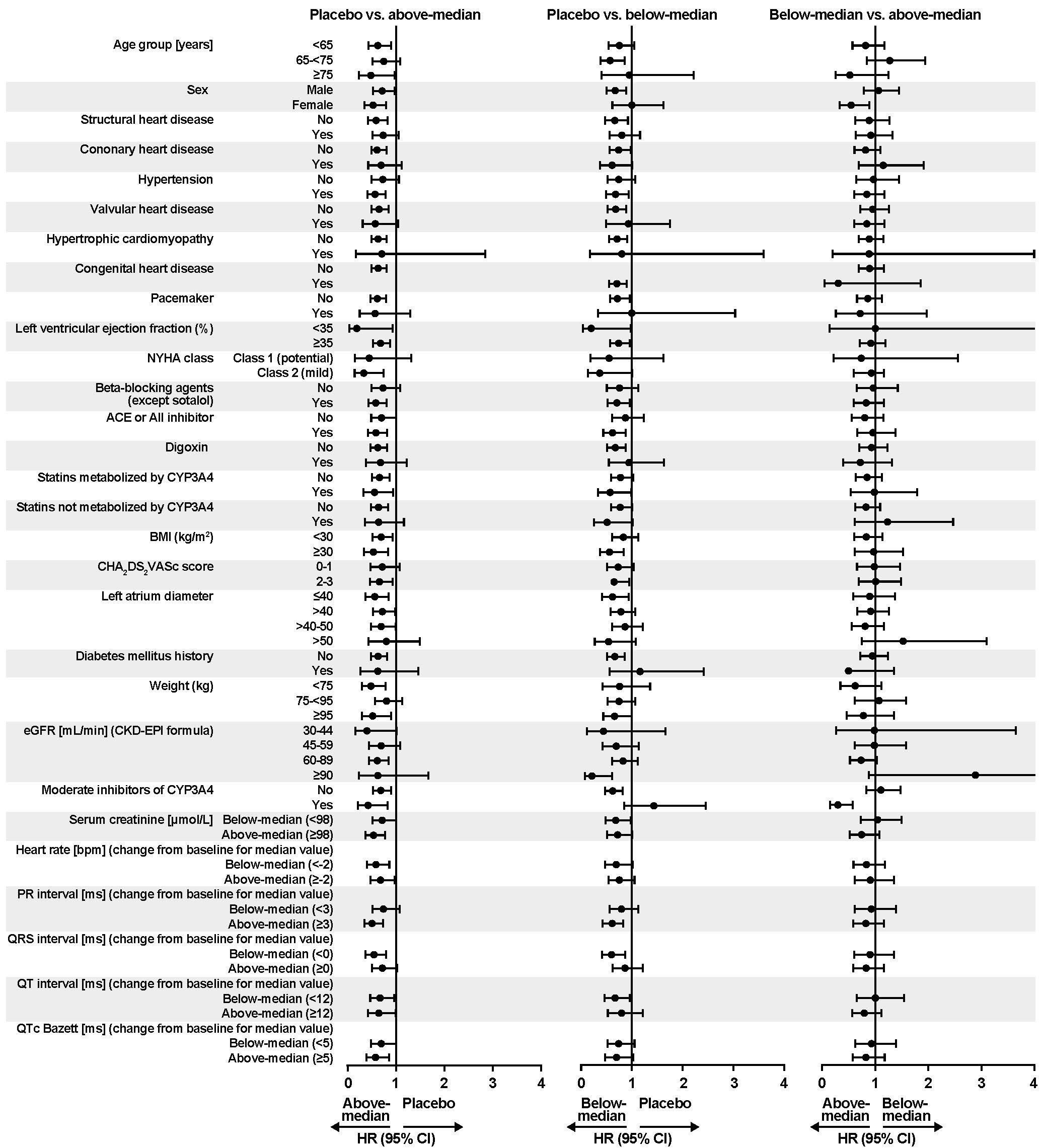


Except for ECG parameters, all data are at baseline.

Abbreviations: ACE, angiotensin-converting enzyme; AF/AFL, atrial fibrillation/atrial flutter; AII, angiotensin II receptor; BMI, body mass index; CI, confidence interval; CKD-EPI, Chronic Kidney Disease Epidemiology Collaboration; C_max_, maximum serum concentration achieved by a drug; C_trough_avg_, average trough concentration; eGFR, estimated glomerular filtration rate; HR, hazard ratio; NYHA, New York Heart Association.

Median dronedarone C­_trough_avg_ = 54 ng/ml; below-median is <54 ng/ml and above-median is ≥54 ng/ml.
